# Comment on Comparison of the Outcome of Low Dose and High-Dose Corticosteroid in the Treatment of Idiopathic Granulomatous Mastitis 

**DOI:** 10.31557/APJCP.2020.21.8.2177

**Published:** 2020-08

**Authors:** Sami Akbulut, Tevfik Tolga Sahin

**Affiliations:** *Department of Surgery and Liver Transplant Institute, Inonu University Faculty of Medicine, 44280, Malatya, Turkey. *

**Dear Editor**

We read with great interest the recent article ‘’Comparison of the Outcome of Low Dose and High-Dose Corticosteroid in the Treatment of Idiopathic Granulomatous Mastitis‘’ published by Montazer et al., (2020). The authors stated that they evaluated the efficacy of low dose vs. high dose prednisolone in the treatment of idiopathic granulomatous mastitis (IGM).

The authors stated that they used prophylactic antibiotherapy before initiation of steroid treatment. There is a general agreement on the initiation of antibiotherapy as the beginning treatment for patients who are admitting with the signs of abscess and cellulitis; we also agree with this approach. However, we do not agree with starting antibiotics to any patient with a breast mass who does not have any signs and symptoms of mastitis. Furthermore, there is no information on whether the authors have started steroid therapy before or after the histopathological examination report. If it is assumed that the biopsy results are not finalized before 10 days; the authors should clarify their management protocol during the waiting period. In the current literature, there is no standard diagnostic and therapeutic algorithm for IGM. This is the main reason for heterogeneity observed in the studies that are published. For this reason, we would like to present our algorithm called the Akbulut-Sahin algorithm for the management of IGM as a result of our experiences and careful analysis of the literature (Figure 1) (Fayed et al., 2019; Wolfrum Yellow et al., 2018; Kaviani et al., 2014; Oran et al., 2013; Seo et al., 2012; Hovanessian Larsen et al., 2009; Wilson et al., 2007).

Also, the authors should give information regarding the total number of patients who were evaluated for possible IGM and were ruled-out to have the disease during the study period. This will give important information regarding the sensitivity and specificity of the clinical suspicion in the diagnosis of IGM. The reason for this is that all of the studies involving IGM were retrospective and non-of them performed such an analysis. In our opinion, the most important information that is missing in the literature is the accuracy of clinical suspicion for the diagnosis of IGM.

Although it shows a wide geographic variance, tuberculosis is very important in the differential diagnosis of IGM. Tuberculosis is endemic in many countries including Iran. For this reason, the patients should be evaluated in detail for tuberculosis for the differential diagnosis of IGM. We believe it would be appropriate if the authors emphasized this point in the present study. 

The authors state that they have used α=0.05, β=0.20, and effect size (≥0.60) for the power analysis and have calculated the minimum sample size to be 15 patients for each group. We used the G*Power software for calculation of the sample size. However, even if we considered the effect size=0.8 the minimum number of subjects required in each group was calculated as 26. Besides, we used the remission variable presented in the authors’ work and calculated the sample size using the MedCalc software and the minimum number of subjects required for the study was 19 for each group. The authors should declare which software they have used for power analysis. 

The authors have compared continuous and categoric variables among low and high dose steroid groups and have presented their data in Table 1. We would like to make a few comments on this analysis. The expected value for categoric variables in the present study is <5 and therefore, Fisher’s Exact test should be used for the analysis. The p-value presented in the recurrence line could not be calculated as 0.04 in any known software. This variable is not significantly different (p=0.224) as authors have stated and this miscalculation will create a bias for the risk factor analysis performed. This point should be clarified for the readers of your journal because the most important point in IGM is the evaluation of the independent risk factors of relapse and recurrence. The authors have neither performed univariate nor multivariate analysis to calculate the odds ratio (OR) which is an important shortcoming of the study. We calculated the OR values using the data provided by the authors (Table 1). We have shown that contrary to what the authors have calculated, the recurrence rate was not statistically significant. Furthermore, the remission rate in the patients who received high-dose steroid was 12.25 times higher than the remission rates in patients with low dose steroid (p=0.035). Also, we found a statistically significant and positive moderate to a strong relationship between the steroid dose and remission (phi=0.452).

**Table 1:** Calculation of Factors Affecting IGM Recurrence Using Univariate and Multivariate Analysis

	Low Dose Steroid	High Dose Steroid	Univariate Analysis			Phi
	(n=15)	(n=15	p	OR	95% CI	
Recurrence (+) *	3 (37.5)	0 (0)	0.224**	8.68	0.41-184	0.333
Remission (+)	8 (53.3)	14 (93.3)	0.035**	12.25	127-118	0.452
Painful mass	10 (66.7)	7 (46.7)	0.461**	2.28	0.52-10	0.202
Pain and erythema	4 (26.7)	5 (33.3)	1.000**	0.72	0.15-3.5	-0.073
Fistula with drainage	1 (6.7)	3 (20)	0.598**	0.29	0.27-3.2	-0.196

*, OR was calculated by Haldane-Anscombe correction analysis; **, Fisher’s Exact

**Figure 1:**
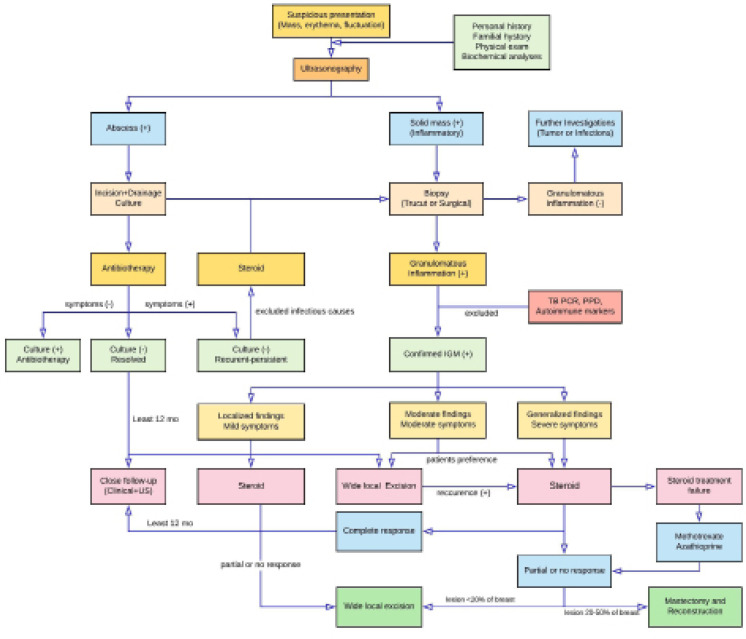
Algorithm for the Diagnostic and Therapeutic Management of Granulomatous Mastitis (Akbulut-Sahin Algorithm for IGM)

## Conflict of interest

The authors declare that they have no conflict of interest.
